# 2-Chloro-*N*-[(4-chloro­phen­yl)(phen­yl)meth­yl]-*N*-[2-(4-nitro-1*H*-imidazol-1-yl)eth­yl]ethanamine

**DOI:** 10.1107/S160053681100256X

**Published:** 2011-01-26

**Authors:** Wen-Tai Zhang, Cheng-He Zhou, Qing-Gang Ji

**Affiliations:** a Laboratory of Bioorganic & Medicinal Chemistry, School of Chemistry and Chemical Engineering, Southwest University, Chongqing 400715, People’s Republic of China

## Abstract

In the title compound, C_20_H_20_Cl_2_N_4_O_2_, the nitro­imidazole ring makes dihedral angles of 17.00 (1) and 60.45 (11)° with the phenyl and chloro­phenyl rings, respectively. The three-coordinate N atom connected to two methyl­ene and one methine C atoms shows pyramidal coordination.

## Related literature

For the use of nitro­gen mustards containing the β-chloro­ethyl­amine unit as anti­tumor drugs, see: Zhuang *et al.* (2008 ▶). Nitro­imidazole compounds are also used extensively in the treatment of various cancers as clinical radiosensitizers, see: Cai *et al.* (2009 ▶). For the synthesis, see: Fang *et al.* (2010 ▶); Gan *et al.* (2010 ▶).
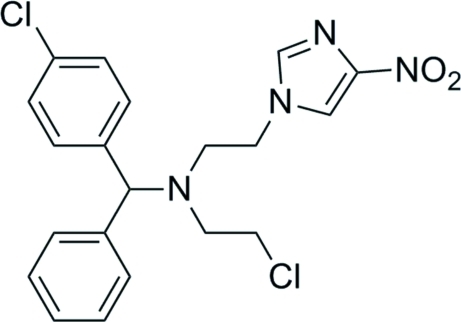


## Experimental

### 

#### Crystal data

C_20_H_20_Cl_2_N_4_O_2_
*M* *_r_* = 419.30
Monoclinic, 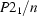

*a* = 8.8206 (16) Å
*b* = 25.005 (5) Å
*c* = 9.0450 (17) Å
β = 100.802 (3)°
*V* = 1959.6 (6) Å^3^
*Z* = 4
Mo *K*α radiation
μ = 0.36 mm^−1^
*T* = 298 K
0.32 × 0.24 × 0.18 mm

#### Data collection

Bruker SMART CCD area-detector diffractometer
Absorption correction: multi-scan (*SADABS*; Sheldrick, 1996 ▶) *T*_min_ = 0.903, *T*_max_ = 0.938
9838 measured reflections
3449 independent reflections
2464 reflections with *I* > 2σ(*I*)
*R*_int_ = 0.037

#### Refinement

*R*[*F* ^2^ > 2σ(*F* ^2^)] = 0.053
*wR*(*F* ^2^) = 0.139
*S* = 1.03
3449 reflections
253 parameters
H-atom parameters constrained
Δρ_max_ = 0.49 e Å^−3^
Δρ_min_ = −0.24 e Å^−3^

### 

Data collection: *SMART* (Bruker, 2000 ▶); cell refinement: *SAINT* (Bruker, 2000 ▶); data reduction: *SAINT*; program(s) used to solve structure: *SHELXS97* (Sheldrick, 2008 ▶); program(s) used to refine structure: *SHELXL97* (Sheldrick, 2008 ▶); molecular graphics: *SHELXTL* (Sheldrick, 2008 ▶); software used to prepare material for publication: *SHELXTL*.

## Supplementary Material

Crystal structure: contains datablocks I, global. DOI: 10.1107/S160053681100256X/ng5107sup1.cif

Structure factors: contains datablocks I. DOI: 10.1107/S160053681100256X/ng5107Isup2.hkl

Additional supplementary materials:  crystallographic information; 3D view; checkCIF report

## Figures and Tables

**Fig. 1.**
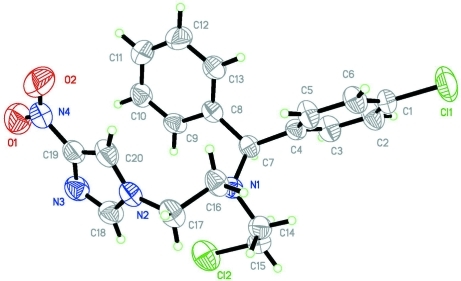
Ellipsoid plot.

## supplementary crystallographic information

### Comment

Nitrogen mustards as anticancer agents containing typical β-chloroethylamine moiety with easy synthesis and inexpensive expense are one of the most important antitumor drugs (Zhuang *et al.*, 2008). Nitroimidazole compounds are also extensively used in the treatment of various cancers as clinical radiosensitizer (Cai *et al.*, 2009). In view of this, it is of great interest for us to investigate the nitrogen mustard-based nitroimidazoles as new potential anticancer agents. Herein we would like to report the crystal structure of the title compound (I).

The title compound, C_20_H_20_Cl_2_N_4_O_2_, crystallized in non-chiral monoclinic crystal system of *P2(1)/n* space group, including a racemic chiral isomers. In the molecule, the nitroimidazole ring makes dihedral angles of 17.00 (1) and 60.45 (11)°, respectively, with the benzene and chlorophenyl ring.

### Experimental

The intermediate 2-chloro-*N*-(2-chloroethyl)-*N*-((4-chlorophenyl(phenyl)methyl)ethanamine (0.85 g, 2.5 mmol), which was prepared according to the procedure of Fang *et al.*(2010) and Gan *et al.*(2010), reacted with 4-nitroimidazole (0.34 g, 3.0 mmol) in the presence of weak base in acetonitrile at 60 °C for 12 h to produce the title compound (I) 0.30 g as white solid *via* silica gel column chromatography (ethyl acetate/petroleum ether, 1/2, V/V). A crystal of (I) suitable for X-ray analysis was grown from a mixture solution of ethyl acetate and petroleum ether by slow evaporation at room temperature.

### Refinement

Hydrogen atoms were placed in idealized positions and treated as riding, with C—H = 0.93Å (CH), 0.98Å (CH) or 0.98Å (CH_2_) and *U*iso(H) = 1.2 *U*eq(C).

### Crystal data

**Table:**

C_20_H_20_Cl_2_N_4_O_2_	*Z* = 4
*M**_r_* = 419.30	*F*(000) = 872
Monoclinic, *P*2_1_/*n*	*D*_x_ = 1.421 Mg m^−^^3^
Hall symbol: -P 2yn	Mo *K*α radiation, λ = 0.71073 Å
*a* = 8.8206 (16) Å	θ = 2.4–21.3°
*b* = 25.005 (5) Å	µ = 0.36 mm^−^^1^
*c* = 9.0450 (17) Å	*T* = 298 K
β = 100.802 (3)°	Block, colourless
*V* = 1959.6 (6) Å^3^	0.32 × 0.24 × 0.18 mm

### Data collection

**Table:**

Bruker SMART CCD area-detector diffractometer	3449 independent reflections
Radiation source: fine-focus sealed tube	2464 reflections with *I* > 2σ(*I*)
graphite	*R*_int_ = 0.037
phi and ω scans	θ_max_ = 25.0°, θ_min_ = 1.6°
Absorption correction: multi-scan (*SADABS*; Sheldrick, 1996)	*h* = −10→7
*T*_min_ = 0.903, *T*_max_ = 0.938	*k* = −29→29
9838 measured reflections	*l* = −10→10

### Refinement

**Table:**

Refinement on *F*^2^	Primary atom site location: structure-invariant direct methods
Least-squares matrix: full	Secondary atom site location: difference Fourier map
*R*[*F*^2^ > 2σ(*F*^2^)] = 0.053	Hydrogen site location: inferred from neighbouring sites
*wR*(*F*^2^) = 0.139	H-atom parameters constrained
*S* = 1.03	*w* = 1/[σ^2^(*F*_o_^2^) + (0.0576*P*)^2^ + 0.904*P*] where *P* = (*F*_o_^2^ + 2*F*_c_^2^)/3
3449 reflections	(Δ/σ)_max_ = 0.001
253 parameters	Δρ_max_ = 0.49 e Å^−^^3^
0 restraints	Δρ_min_ = −0.24 e Å^−^^3^

### Special details

**Table:**

Geometry. All e.s.d.'s (except the e.s.d. in the dihedral angle between two l.s. planes) are estimated using the full covariance matrix. The cell e.s.d.'s are taken into account individually in the estimation of e.s.d.'s in distances, angles and torsion angles; correlations between e.s.d.'s in cell parameters are only used when they are defined by crystal symmetry. An approximate (isotropic) treatment of cell e.s.d.'s is used for estimating e.s.d.'s involving l.s. planes.
Refinement. Refinement of *F*^2^ against ALL reflections. The weighted *R*-factor *wR* and goodness of fit *S* are based on *F*^2^, conventional *R*-factors *R* are based on *F*, with *F* set to zero for negative *F*^2^. The threshold expression of *F*^2^ > σ(*F*^2^) is used only for calculating *R*-factors(gt) *etc*. and is not relevant to the choice of reflections for refinement. *R*-factors based on *F*^2^ are statistically about twice as large as those based on *F*, and *R*- factors based on ALL data will be even larger.

### Fractional atomic coordinates and isotropic or equivalent isotropic displacement parameters (Å^2^)

**Table:**

	*x*	*y*	*z*	*U*_iso_*/*U*_eq_	
C1	0.3270 (3)	0.05952 (13)	0.0927 (3)	0.0487 (7)	
C2	0.4173 (4)	0.01834 (13)	0.1569 (4)	0.0615 (9)	
H2	0.3972	−0.0165	0.1231	0.074*	
C3	0.5385 (4)	0.02915 (12)	0.2723 (3)	0.0538 (8)	
H3	0.5994	0.0009	0.3161	0.065*	
C4	0.5734 (3)	0.07989 (11)	0.3257 (3)	0.0402 (6)	
C5	0.4771 (3)	0.12070 (12)	0.2588 (3)	0.0498 (7)	
H5	0.4957	0.1556	0.2933	0.060*	
C6	0.3544 (3)	0.11081 (12)	0.1423 (3)	0.0514 (8)	
H6	0.2917	0.1386	0.0985	0.062*	
C7	0.7143 (3)	0.08861 (11)	0.4496 (3)	0.0401 (6)	
H7	0.7628	0.0533	0.4654	0.048*	
C8	0.6827 (3)	0.10452 (10)	0.6032 (3)	0.0400 (6)	
C9	0.8038 (3)	0.10174 (11)	0.7245 (3)	0.0456 (7)	
H9	0.8999	0.0902	0.7091	0.055*	
C10	0.7859 (4)	0.11550 (12)	0.8666 (3)	0.0536 (8)	
H10	0.8695	0.1137	0.9463	0.064*	
C11	0.6439 (4)	0.13203 (13)	0.8913 (4)	0.0592 (8)	
H11	0.6313	0.1417	0.9875	0.071*	
C12	0.5226 (4)	0.13412 (14)	0.7746 (4)	0.0657 (9)	
H12	0.4263	0.1448	0.7912	0.079*	
C13	0.5415 (3)	0.12044 (12)	0.6309 (3)	0.0538 (8)	
H13	0.4573	0.1220	0.5518	0.065*	
C14	0.9049 (3)	0.10277 (13)	0.2887 (3)	0.0547 (8)	
H14A	0.9671	0.1308	0.2559	0.066*	
H14B	0.8231	0.0941	0.2046	0.066*	
C15	1.0037 (4)	0.05433 (13)	0.3272 (4)	0.0591 (8)	
H15A	1.0457	0.0436	0.2400	0.071*	
H15B	0.9407	0.0252	0.3526	0.071*	
C16	0.7954 (3)	0.18032 (11)	0.3913 (3)	0.0498 (7)	
H16A	0.7132	0.1884	0.4456	0.060*	
H16B	0.7574	0.1876	0.2855	0.060*	
C17	0.9333 (4)	0.21643 (13)	0.4485 (3)	0.0557 (8)	
H17A	1.0205	0.2049	0.4051	0.067*	
H17B	0.9081	0.2528	0.4158	0.067*	
C18	1.1030 (3)	0.19238 (12)	0.6938 (4)	0.0523 (8)	
H18	1.1775	0.1748	0.6516	0.063*	
C19	0.9802 (3)	0.22472 (11)	0.8478 (3)	0.0479 (7)	
C20	0.8953 (3)	0.23602 (11)	0.7104 (4)	0.0521 (8)	
H20	0.8017	0.2542	0.6891	0.063*	
Cl1	0.17478 (11)	0.04618 (4)	−0.05458 (11)	0.0835 (3)	
Cl2	1.15787 (10)	0.06636 (4)	0.48090 (12)	0.0777 (3)	
N1	0.8356 (2)	0.12352 (9)	0.4102 (2)	0.0412 (5)	
N2	0.9760 (3)	0.21524 (9)	0.6115 (3)	0.0458 (6)	
N3	1.1106 (3)	0.19743 (10)	0.8388 (3)	0.0538 (6)	
N4	0.9403 (4)	0.23746 (13)	0.9901 (4)	0.0692 (8)	
O1	1.0250 (3)	0.22177 (12)	1.1050 (3)	0.0897 (9)	
O2	0.8228 (4)	0.26290 (15)	0.9864 (4)	0.1142 (12)	

### Atomic displacement parameters (Å^2^)

**Table:**

	*U*^11^	*U*^22^	*U*^33^	*U*^12^	*U*^13^	*U*^23^
C1	0.0397 (16)	0.0603 (19)	0.0430 (17)	−0.0055 (14)	−0.0002 (13)	−0.0095 (14)
C2	0.059 (2)	0.0473 (18)	0.073 (2)	−0.0065 (16)	−0.0015 (18)	−0.0138 (16)
C3	0.0534 (18)	0.0446 (17)	0.060 (2)	0.0017 (14)	0.0013 (16)	−0.0014 (14)
C4	0.0360 (14)	0.0425 (15)	0.0416 (16)	0.0020 (12)	0.0059 (12)	0.0013 (12)
C5	0.0451 (16)	0.0416 (16)	0.0565 (19)	0.0023 (13)	−0.0061 (14)	−0.0055 (14)
C6	0.0427 (17)	0.0503 (18)	0.0561 (19)	0.0063 (14)	−0.0040 (14)	0.0038 (14)
C7	0.0361 (14)	0.0399 (15)	0.0417 (16)	0.0039 (12)	0.0005 (12)	0.0038 (12)
C8	0.0383 (15)	0.0369 (14)	0.0433 (16)	−0.0022 (12)	0.0041 (12)	0.0054 (12)
C9	0.0347 (15)	0.0561 (18)	0.0450 (17)	0.0012 (13)	0.0049 (13)	0.0102 (14)
C10	0.0498 (18)	0.068 (2)	0.0392 (17)	−0.0077 (16)	−0.0004 (14)	0.0072 (15)
C11	0.063 (2)	0.068 (2)	0.0475 (18)	−0.0007 (17)	0.0118 (16)	−0.0073 (16)
C12	0.0505 (19)	0.087 (3)	0.059 (2)	0.0159 (18)	0.0107 (17)	−0.0067 (18)
C13	0.0397 (16)	0.069 (2)	0.0493 (18)	0.0076 (15)	−0.0010 (14)	−0.0013 (15)
C14	0.0442 (17)	0.076 (2)	0.0419 (17)	−0.0036 (16)	0.0032 (14)	0.0063 (15)
C15	0.0513 (19)	0.072 (2)	0.057 (2)	−0.0036 (17)	0.0181 (16)	−0.0093 (17)
C16	0.0454 (17)	0.0521 (18)	0.0463 (17)	−0.0048 (14)	−0.0055 (14)	0.0111 (14)
C17	0.0549 (19)	0.0574 (19)	0.0500 (18)	−0.0139 (15)	−0.0022 (15)	0.0171 (15)
C18	0.0396 (16)	0.0621 (19)	0.054 (2)	0.0032 (15)	0.0060 (14)	0.0025 (15)
C19	0.0413 (16)	0.0467 (17)	0.0552 (19)	−0.0076 (14)	0.0074 (14)	−0.0049 (14)
C20	0.0404 (17)	0.0441 (17)	0.067 (2)	0.0010 (14)	−0.0030 (16)	−0.0054 (15)
Cl1	0.0626 (6)	0.0995 (7)	0.0751 (6)	−0.0043 (5)	−0.0216 (5)	−0.0236 (5)
Cl2	0.0537 (5)	0.0773 (6)	0.0937 (7)	0.0113 (4)	−0.0080 (5)	0.0054 (5)
N1	0.0341 (12)	0.0473 (13)	0.0411 (13)	0.0014 (10)	0.0038 (10)	0.0070 (10)
N2	0.0381 (13)	0.0465 (13)	0.0494 (15)	−0.0064 (11)	−0.0001 (11)	0.0047 (11)
N3	0.0452 (15)	0.0627 (16)	0.0507 (16)	−0.0013 (13)	0.0017 (12)	0.0051 (13)
N4	0.0560 (19)	0.080 (2)	0.072 (2)	−0.0215 (16)	0.0145 (17)	−0.0256 (17)
O1	0.094 (2)	0.117 (2)	0.0570 (16)	−0.0273 (18)	0.0125 (16)	−0.0085 (15)
O2	0.078 (2)	0.151 (3)	0.117 (3)	0.012 (2)	0.0262 (18)	−0.065 (2)

### Geometric parameters (Å, °)

**Table:**

C1—C2	1.364 (4)	C13—H13	0.9300
C1—C6	1.365 (4)	C14—N1	1.450 (4)
C1—Cl1	1.737 (3)	C14—C15	1.495 (4)
C2—C3	1.374 (4)	C14—H14A	0.9700
C2—H2	0.9300	C14—H14B	0.9700
C3—C4	1.372 (4)	C15—Cl2	1.779 (3)
C3—H3	0.9300	C15—H15A	0.9700
C4—C5	1.392 (4)	C15—H15B	0.9700
C4—C7	1.524 (4)	C16—N1	1.466 (3)
C5—C6	1.384 (4)	C16—C17	1.525 (4)
C5—H5	0.9300	C16—H16A	0.9700
C6—H6	0.9300	C16—H16B	0.9700
C7—N1	1.475 (3)	C17—N2	1.452 (4)
C7—C8	1.520 (4)	C17—H17A	0.9700
C7—H7	0.9800	C17—H17B	0.9700
C8—C13	1.375 (4)	C18—N3	1.307 (4)
C8—C9	1.382 (4)	C18—N2	1.350 (4)
C9—C10	1.368 (4)	C18—H18	0.9300
C9—H9	0.9300	C19—N3	1.353 (4)
C10—C11	1.376 (4)	C19—C20	1.355 (4)
C10—H10	0.9300	C19—N4	1.432 (4)
C11—C12	1.356 (4)	C20—N2	1.348 (4)
C11—H11	0.9300	C20—H20	0.9300
C12—C13	1.384 (4)	N4—O2	1.211 (4)
C12—H12	0.9300	N4—O1	1.225 (4)
			
C2—C1—C6	121.1 (3)	N1—C14—H14A	108.5
C2—C1—Cl1	119.2 (2)	C15—C14—H14A	108.5
C6—C1—Cl1	119.8 (2)	N1—C14—H14B	108.5
C1—C2—C3	119.0 (3)	C15—C14—H14B	108.5
C1—C2—H2	120.5	H14A—C14—H14B	107.5
C3—C2—H2	120.5	C14—C15—Cl2	111.8 (2)
C4—C3—C2	122.8 (3)	C14—C15—H15A	109.2
C4—C3—H3	118.6	Cl2—C15—H15A	109.2
C2—C3—H3	118.6	C14—C15—H15B	109.2
C3—C4—C5	116.5 (3)	Cl2—C15—H15B	109.2
C3—C4—C7	119.3 (2)	H15A—C15—H15B	107.9
C5—C4—C7	124.2 (2)	N1—C16—C17	112.0 (2)
C6—C5—C4	121.8 (3)	N1—C16—H16A	109.2
C6—C5—H5	119.1	C17—C16—H16A	109.2
C4—C5—H5	119.1	N1—C16—H16B	109.2
C1—C6—C5	118.9 (3)	C17—C16—H16B	109.2
C1—C6—H6	120.6	H16A—C16—H16B	107.9
C5—C6—H6	120.6	N2—C17—C16	111.8 (2)
N1—C7—C8	109.3 (2)	N2—C17—H17A	109.3
N1—C7—C4	115.8 (2)	C16—C17—H17A	109.3
C8—C7—C4	116.5 (2)	N2—C17—H17B	109.3
N1—C7—H7	104.6	C16—C17—H17B	109.3
C8—C7—H7	104.6	H17A—C17—H17B	107.9
C4—C7—H7	104.6	N3—C18—N2	113.2 (3)
C13—C8—C9	117.5 (3)	N3—C18—H18	123.4
C13—C8—C7	124.7 (3)	N2—C18—H18	123.4
C9—C8—C7	117.8 (2)	N3—C19—C20	112.3 (3)
C10—C9—C8	121.7 (3)	N3—C19—N4	121.4 (3)
C10—C9—H9	119.2	C20—C19—N4	126.2 (3)
C8—C9—H9	119.2	N2—C20—C19	105.0 (3)
C9—C10—C11	119.8 (3)	N2—C20—H20	127.5
C9—C10—H10	120.1	C19—C20—H20	127.5
C11—C10—H10	120.1	C14—N1—C16	112.7 (2)
C12—C11—C10	119.7 (3)	C14—N1—C7	113.5 (2)
C12—C11—H11	120.2	C16—N1—C7	115.5 (2)
C10—C11—H11	120.2	C20—N2—C18	106.5 (2)
C11—C12—C13	120.4 (3)	C20—N2—C17	126.7 (3)
C11—C12—H12	119.8	C18—N2—C17	126.8 (3)
C13—C12—H12	119.8	C18—N3—C19	103.0 (3)
C8—C13—C12	121.0 (3)	O2—N4—O1	125.0 (3)
C8—C13—H13	119.5	O2—N4—C19	116.4 (3)
C12—C13—H13	119.5	O1—N4—C19	118.5 (3)
N1—C14—C15	115.1 (2)		
			
C6—C1—C2—C3	−0.6 (5)	N1—C14—C15—Cl2	−58.3 (3)
Cl1—C1—C2—C3	179.4 (2)	N1—C16—C17—N2	−70.4 (3)
C1—C2—C3—C4	−0.4 (5)	N3—C19—C20—N2	−0.3 (3)
C2—C3—C4—C5	1.3 (4)	N4—C19—C20—N2	−178.3 (3)
C2—C3—C4—C7	−177.3 (3)	C15—C14—N1—C16	156.5 (3)
C3—C4—C5—C6	−1.3 (4)	C15—C14—N1—C7	−69.7 (3)
C7—C4—C5—C6	177.2 (3)	C17—C16—N1—C14	−83.4 (3)
C2—C1—C6—C5	0.6 (5)	C17—C16—N1—C7	143.8 (2)
Cl1—C1—C6—C5	−179.4 (2)	C8—C7—N1—C14	163.5 (2)
C4—C5—C6—C1	0.4 (5)	C4—C7—N1—C14	−62.6 (3)
C3—C4—C7—N1	118.5 (3)	C8—C7—N1—C16	−64.1 (3)
C5—C4—C7—N1	−60.0 (3)	C4—C7—N1—C16	69.8 (3)
C3—C4—C7—C8	−110.9 (3)	C19—C20—N2—C18	0.6 (3)
C5—C4—C7—C8	70.6 (3)	C19—C20—N2—C17	179.6 (3)
N1—C7—C8—C13	121.7 (3)	N3—C18—N2—C20	−0.7 (3)
C4—C7—C8—C13	−11.9 (4)	N3—C18—N2—C17	−179.7 (3)
N1—C7—C8—C9	−59.8 (3)	C16—C17—N2—C20	−70.0 (4)
C4—C7—C8—C9	166.6 (2)	C16—C17—N2—C18	108.8 (3)
C13—C8—C9—C10	−1.6 (4)	N2—C18—N3—C19	0.4 (3)
C7—C8—C9—C10	179.7 (3)	C20—C19—N3—C18	−0.1 (3)
C8—C9—C10—C11	0.7 (4)	N4—C19—N3—C18	178.0 (3)
C9—C10—C11—C12	0.5 (5)	N3—C19—N4—O2	178.6 (3)
C10—C11—C12—C13	−0.9 (5)	C20—C19—N4—O2	−3.6 (5)
C9—C8—C13—C12	1.2 (4)	N3—C19—N4—O1	−2.0 (4)
C7—C8—C13—C12	179.8 (3)	C20—C19—N4—O1	175.8 (3)
C11—C12—C13—C8	0.0 (5)		
